# The Antiviral Mechanism of an Influenza A Virus Nucleoprotein-Specific Single-Domain Antibody Fragment

**DOI:** 10.1128/mBio.01569-16

**Published:** 2016-12-13

**Authors:** Leo Hanke, Kevin E. Knockenhauer, R. Camille Brewer, Eline van Diest, Florian I. Schmidt, Thomas U. Schwartz, Hidde L. Ploegh

**Affiliations:** a Whitehead Institute for Biomedical Research, Cambridge, Massachusetts, USA; b Department of Biology, Massachusetts Institute of Technology, Cambridge, Massachusetts, USA

## Abstract

Alpaca-derived single-domain antibody fragments (VHHs) that target the influenza A virus nucleoprotein (NP) can protect cells from infection when expressed in the cytosol. We found that one such VHH, αNP-VHH1, exhibits antiviral activity similar to that of Mx proteins by blocking nuclear import of incoming viral ribonucleoproteins (vRNPs) and viral transcription and replication in the nucleus. We determined a 3.2-Å crystal structure of αNP-VHH1 in complex with influenza A virus NP. The VHH binds to a nonconserved region on the body domain of NP, which has been associated with binding to host factors and serves as a determinant of host range. Several of the NP/VHH interface residues determine sensitivity of NP to antiviral Mx GTPases. The structure of the NP/αNP-VHH1 complex affords a plausible explanation for the inhibitory properties of the VHH and suggests a rationale for the antiviral properties of Mx proteins. Such knowledge can be leveraged for much-needed novel antiviral strategies.

## IMPORTANCE

Influenza virus strains can rapidly escape from protection afforded by seasonal vaccines or acquire resistance to available drugs. Additional ways to interfere with the virus life cycle are therefore urgently needed. The influenza virus nucleoprotein is one promising target for antiviral interventions. We have previously isolated alpaca-derived single-domain antibody fragments (VHHs) that protect cells from influenza virus infection if expressed intracellularly. We show here that one such VHH exhibits antiviral activities similar to those of proteins of the cellular antiviral defense (Mx proteins). We determined the three-dimensional structure of this VHH in complex with the influenza virus nucleoprotein and identified the interaction site, which overlaps regions that determine sensitivity of the virus to Mx proteins. Our data define a new vulnerability of influenza virus, help us to better understand the cellular antiviral mechanisms, and provide a well-characterized tool to further study them.

## INTRODUCTION

Seasonal human influenza A virus (IAV) epidemics and occasional pandemics cause significant morbidity and continue to pose a large economic burden. Influenza A virus is a segmented, negative-stranded RNA virus and belongs to the family *Orthomyxoviridae*. To infect a cell, virus particles must fuse with the host cell membrane to release the viral ribonucleoprotein (vRNP) complexes into the cytosol. vRNPs must then cross the nuclear membrane to enter the nucleus, where viral transcription and replication take place.

vRNPs contain a negative-stranded RNA genome segment decorated by many copies of the nucleoprotein (NP). NP bound to viral RNA (vRNA) assembles into two helical, antiparallel strands that form a loop at one end. At the other end, the heterotrimeric polymerase, comprised of PA, PB1, and PB2, is positioned and binds to the base-paired, conserved ends of the vRNA as well as to NP.

The major vRNP component, NP, is a basic, ~56-kDa protein consisting of a head and body domain and a tail loop (amino acids [aa] 402 to 428). In the context of a vRNP, the tail loop protrudes into adjacent NP molecules and mediates oligomerization (1). Besides binding and condensing vRNA, the diverse functions of NP include nuclear import, export, and RNA synthesis. NP interacts with the viral proteins M1 and PB2 and a variety of host proteins (2–6). Because of its complex interactions, NP is a key determinant of host specificity, such that IAV strains with NP sequences of avian origin are much less virulent in human cells (7, 8).

Nuclear import of vRNPs through nuclear pores is mediated by importin-α/β. At least two nuclear localization sequences (NLSs) that are recognized by the different importin-α isoforms are present on NP, including a nonconventional NLS (NLS1, NP residues 3 to 13) and NLS2, comprising residues 198 to 216 (9, 10). NP is dispensable for transcription of short (<100 nucleotides [nt]) vRNA-like templates but is essential for transcription and replication of full-length viral genome segments. NP thus serves as a crucial factor for transcript elongation (11). Newly synthesized NP exists as monomers and small oligomers (trimers) and is imported into the nucleus to assemble new vRNPs (12). Later in infection, the progeny vRNPs are exported to the cytosol with the aid of the viral nuclear export protein (NEP), M1, and the host CRM1 export machinery for delivery to the budding site (5, 13).

Anti-influenza virus drugs and vaccines have traditionally targeted the surface-exposed viral hemagglutinin (HA), neuraminidase (NA), or the ion channel M2. The selective pressure exerted by drugs, antibodies, and T cells confers a selective advantage on those viruses with mutations in their surface proteins as a means of escape (antigenic drift). Strain-dependent sequence variation in other influenza virus proteins, such as NP, exists but is far less prominent than the antigenic drift seen for HA, NA, and M2 variants.

Alternative approaches to traditional interventions must therefore target the more conserved proteins of the viral RNP complex to prevent them from entering the nucleus or to inhibit the associated activity of the RNA-dependent RNA polymerase, a vulnerability of the virus that is also exploited by host antiviral Mx proteins. Mx GTPases are interferon-induced effectors of the cell-autonomous antiviral immune response, with broad specificity against a number of RNA viruses. Although molecular details of how Mx proteins exert their antiviral activity are unknown, we do know the stages in the viral life cycle that are perturbed by Mx proteins (14). The human MxA protein is localized in the cytosol and prevents nuclear import of incoming vRNPs (15). In contrast, mouse Mx1, a close homolog, is localized in the nucleus and interferes with viral transcription and replication (14).

To better relate specific contributions of different NP surface regions to their function and to identify new vulnerabilities that perturb the viral life cycle, we have used intracellularly expressed variable domains of alpaca heavy-chain-only antibodies (VHHs) that target IAV NP. In contrast to conventional antibodies or their fragments, camelid heavy-chain-only antibodies recognize their target with a single variable binding domain. As fragments, these ~15-kDa antibody domains can bind with high affinity and specificity and are soluble and mostly independent of stabilization by disulfide bonds. This allows expression of VHHs in the cytosol of mammalian cells with retention of their binding properties. Intracellular expression of the anti-NP VHHs during IAV infection disrupts the viral replication cycle at an early stage by preventing essential nuclear import of incoming vRNPs but not of NP alone (16, 17). We can thus target a specific NP surface and examine the consequences for virus replication.

We found that one of our NP-specific VHHs, αNP-VHH1, blocks vRNP nuclear import, viral transcription, and replication in a similar fashion as do interferon-induced, antiviral Mx proteins. We used VHHs as crystallization chaperones to grow crystals of recombinant NP in complex with αNP-VHH1. The 3.2-Å crystal structure reveals that the VHH binding site overlaps a region associated with viral sensitivity to Mx proteins. According to one of the proposed vRNP structures, VHH binding occludes access to NLS2 on the adjacent NP molecule, thus providing a plausible mechanistic explanation for the antiviral activity of αNP-VHH1 and Mx proteins.

## RESULTS

### αNP-VHH1 inhibits virus replication after nuclear import of vRNPs.

**FIG 1:**
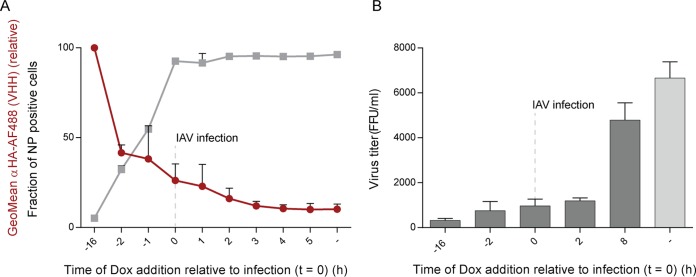
αNP-VHH1 impairs influenza A virus replication at early and late time points. A549 cells expressing αNP-VHH1-HA in a doxycycline (Dox)-inducible manner were seeded 24 h before influenza A virus (IAV) infection. VHH expression was induced at the indicated time points relative to infection; cells were infected with IAV at an MOI of 1 at *t* = 0 h. (A) Cells were harvested 6 h postinfection (p.i.), stained for HA and NP, and analyzed by flow cytometry. Geometric mean of anti-HA-Alexa Fluor 488 (VHH expression level, red) and fraction of infected cells (NP positive, gray) are shown. Mean values ± standard deviations from technical duplicates are displayed. (B) Supernatants were collected 24 h p.i., and titers were determined on MDCK cells. Twenty-four hours p.i., NP in infected MDCK cells (nuclei) was stained with αNP-VHH2-TAMRA, and infected cells were quantified by CellProfiler. Data from three independent experiments (± standard errors of the means) are shown.

We reported elsewhere the identification of several VHHs that target influenza virus NP (16, 17). When expressed in the cytosol, almost all identified VHHs block nuclear import of incoming vRNPs. Due to the multiple roles of NP in the viral life cycle, it is likely that VHHs interfere with virus replication at multiple steps. We tested the effect of αNP-VHH1 in the course of an influenza virus infection in A549 cell derivatives in which VHH expression is doxycycline inducible. Since αNP-VHH1 inhibits the initial nuclear import of vRNPs, we infected cells with influenza virus and induced expression of αNP-VHH1 in the cytosol at different time points from 16 h before to 5 h after infection. We expected that induction of VHH expression after infection would permit vRNP import into the nucleus, which would then allow us to examine the effects of αNP-VHH1 after this step. We assessed expression levels of NP and HA-tagged anti-NP-VHH1 by flow cytometry 6 h postinfection (p.i.), using anti-NP-VHH2 Alexa Fluor (AF) 647 (another NP-specific VHH) and an Alexa Fluor 488-labeled monoclonal anti-HA antibody, respectively (Fig. 1A). VHH levels were lower when their expression was induced at later time points, while the fraction of NP-positive cells and thus infection was higher. When expression was induced 3 or 4 h p.i., VHH levels were barely detectable, which we attribute to influenza virus-mediated shutdown of host protein synthesis (18). Induction of VHH expression as early as 1 h prior to infection was sufficient to prevent most *de novo* NP production, likely by limiting access of vRNPs to the nucleus. A substantial fraction of cells still expressed NP when doxycycline was added simultaneously with infection or 2 h p.i. This leaves a narrow window to allow VHH expression in infected cells to study VHH perturbation after nuclear import. To evaluate the effect of the small quantities of VHH induced at 0 and 2 h p.i., we used the same experimental setup as before but instead quantified virus titers in the supernatants (Fig. 1B). Time points that allowed both VHH production and NP synthesis (0 and 2 h p.i.) yielded drastically reduced virus titers. This suggests that αNP-VHH1 impairs viral replication in ways other than its interference with nuclear import of incoming vRNPs.

### αNP-VHH1 inhibits replication/transcription of long RNA segments.

**FIG 2:**
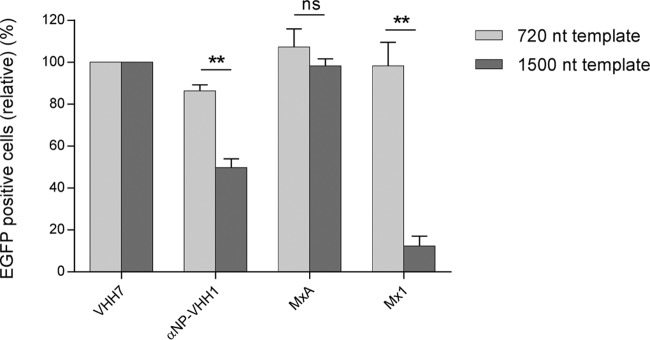
Inhibition of polymerase activity by αNP-VHH1 and Mx1 is dependent on transcript length. 293T cells were transfected with expression vectors for influenza virus A/WSN/33 PA, PB1, PB2, and NP, as well as the indicated VHHs or Mx proteins. In addition, we cotransfected plasmid pPolI-RT, from which a synthetic genome segment was transcribed which encoded either EGFP (720 nt) or mCherry-T2A-EGFP (1,500 nt). Twenty-four hours posttransfection, EGFP-positive cells were quantified by flow cytometry. Since reduced EGFP levels were expressed from the mCherry-T2A-EGFP construct, values were normalized to EGFP-positive cells expressing VHH7 (control). Data from three independent experiments are shown (± standard errors of the means). Statistical significance was assessed by Student’s *t* test (**, *P* < 0.01; ns, not significant).

Nuclear import of vRNPs is followed by transcription and replication of viral genome segments. We have previously analyzed polymerase activity in the presence of NP-specific VHHs using a transfection-based polymerase reconstitution assay in 293T cells, bypassing natural infection and nuclear import of vRNPs. We found that αNP-VHH1 fused to mCherry blocked the synthesis of the template genome encoding the viral NA (16), but HA-tagged αNP-VHH1 did not affect transcription of an artificial genome segment encoding enhanced green fluorescent protein (EGFP) in independent experiments (17). We excluded the possibility that the size of the VHH fusions contributed to this discrepancy (data not shown). Since the template genome segments that we used differ in their length, we speculated that the VHH may interfere with the role of NP in transcript elongation. An interference with elongation that is dependent on the length of the viral genome segment is also seen for antiviral Mx proteins located in the nucleus (14). Initiation and synthesis of primary viral transcripts for M1 and NS2 are barely affected by Mx1, but Mx1 interferes with the synthesis of the longer transcripts encoding NP, HA, PA, PB2, or PB1 (14). To test whether the inhibitory effect of αNP-VHH1 during transcription and replication is dependent on the length of the transcript, we designed two artificial genome segments: one encoding EGFP on a 720-nucleotide (nt) transcript, the other encoding mCherry-T2A-EGFP on a 1,500-nt transcript. This approach enabled us to compare templates of different lengths in a polymerase reconstitution assay, while measuring the same fluorescent molecule as a readout (EGFP). We cotransfected a control VHH (VHH7, anti-murine major histocompatibility complex class II [MHCII]), αNP-VHH1, human MxA, or murine Mx1 and quantified EGFP-positive cells 24 h posttransfection (Fig. 2). The fraction of EGFP-positive cells in the presence of all tested proteins was unaffected for the short 720-nt template, at least at the cotransfected amounts of plasmid DNA. The 1,500-nt genome segment showed reduced overall expression compared to the 720-nt genome segment in the absence of any perturbants. Cotransfection of Mx1 or αNP-VHH1 clearly reduced EGFP-positive cells and thus polymerase efficiency on the 1,500-nt transcript, while the control VHH and MxA did not affect EGFP expression. We thus show that, similarly to Mx proteins in the nucleus, αNP-VHH1 interacts with NP in a manner that does not block initiation of polymerase activity but rather hinders NP in its role as a factor for transcript elongation by the RNA-dependent polymerase. This also explains the reduced virus titers in the presence of anti-NP-VHH1, although we cannot exclude the possibility that additional stages of the replication cycle, such as nuclear export, transport to the budding site, and virion assembly, are impaired by the VHH as well.

### αNP-VHH1 binds to the NP body domain.

**FIG 3:**
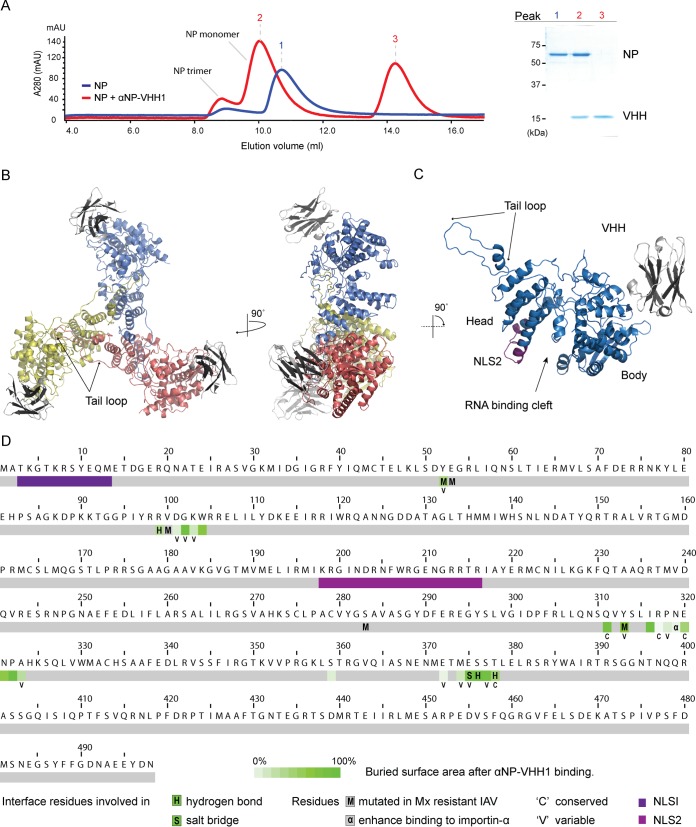
αNP-VHH1 binds to the body domain of NP. (A) NP alone (blue) or preincubated with an excess of αNP-VHH1 (red) was subjected to size exclusion chromatography with a Superdex 200 column. Absorbance at 280 nm of the elution profile is displayed (left). Samples of the peak fractions 1, 2, and 3 were analyzed by SDS-PAGE and Coomassie blue staining (right). (B and C) Ribbon representation of αNP-VHH1 in complex with NP. (B) The three assembled NP molecules that form the trimer are shown in yellow, red, and blue; the VHH (gray) is bound opposite the tail loop. (C) NP monomer bound by αNP-VHH1. NP subdomains, RNA binding cleft, and NLS2 are indicated. NLS1 is disordered in the electron density maps and not shown. All illustrations were generated in PyMOL. (D) Sequence of influenza virus A/WSN/33 NP. The NP/αNP-VHH1 interface residues are shown in green. “H” labels residues involved in hydrogen bonds, and “S” labels residues engaged in the salt bridge at the αNP-VHH1 interface. Conserved interface residues are marked with a “C” (conservation grades 8 to 9), and variable interface residues are marked with a “V” (conservation grades 1 to 3) according to the work of Kukol and Hughes (24). Unlabeled interface residues exhibit average conservation grades. The two nuclear localization sequences (NLSs) of NP are shown in purple and magenta. Residues associated with sensitivity of influenza A virus to Mx proteins are indicated with an “M” (31–33); residues enhancing importin-α binding are indicated with “α” (25).

To define the molecular binding site of αNP-VHH1 on NP as a means of obtaining mechanistic insight into its inhibitory properties, we determined the binding site by X-ray crystallography. To produce the NP/αNP-VHH1 complex, we expressed and purified both proteins individually. We then combined the proteins in a 1:3 NP/αNP-VHH1 molar ratio and purified the complex by size exclusion chromatography. In the absence of RNA, purified NP exists as monomers or trimers, and the oligomerization state can be influenced by salt concentration (19). As in vRNPs, the NP-NP interaction in trimers is facilitated by the NP tail loop that protrudes into an adjacent NP molecule (20). In the size exclusion elution profile, both monomeric and trimeric peaks shift after addition of the VHH, indicating that binding of the VHH does not affect NP oligomerization and that αNP-VHH1 can interact with both species (Fig. 3A).

We obtained cocrystals of the complex that diffracted to 3.2 Å (Table 1) and solved the structure by molecular replacement (MR) using the available structures of NP (PDB identifier [ID] 2IQH) and a VHH (PDB ID 4KRL) as reference models (1, 21). Our model was refined to a final *R*_work_ of 20.4% and *R*_free_ of 26.1%. The NP/αNP-VHH1 complex resembles previously characterized NP structures, forming a trimer with the tail loops projecting into the adjacent NP molecule (Fig. 3B) (1, 22). Anti-NP-VHH1 binds to the body domain at the end opposite the tail loop of each NP monomer (Fig. 3B and C), underlining that it does not interfere with oligomerization of NP. Despite being a potent inhibitor of vRNP nuclear import, the VHH binding site is distant from the known NLS1 and NLS2 (Fig. 3C and D). It is also not in the proximity of the RNA binding cleft (Fig. 3C) and thus likely does not alter binding of NP to RNA.

**TABLE 1:** Data collection and refinement statistics

Characteristic	Influenza A virus NP, αNP-VHH1 native
Protein	
Organism	Influenza A virus*, Vicugna pacos*
PDB ID	5TJW
Data collection	
Space group	P2_1_3
a, b, c (Å)	137.548, 137.548, 137.548
α, β, γ (°)	90.0, 90.0, 90.0
Wavelength (Å)	0.9778
Resolution range (Å)	97.26–3.22 (3.34–3.22)^a^
No. of total reflections	132,024
No. of unique reflections	14,205
Completeness (%)	99.9 (100.0)
Redundancy	9.3 (7.9)
*R*_sym_ (%)	17.8 (100.0)
*R*_p.i.m._ (%)	6.6 (44.2)
I/σ	15.4 (2.1)
CC_1/2_ (%)	99.6 (68.7)
Refinement	
Resolution range (Å)	97.26–3.23
*R*_work_ (%)	20.44
*R*_free_ (%)	26.16
Coordinate error (Å)	0.38
No. of reflections	
Total	14,189
*R*_free_ reflections	1,419
No. of nonhydrogen atoms	4,579
No. of protein atoms	4,579
Root mean square deviations	
Bond lengths (Å)	0.005
Bond angles (°)	0.63
Avg B factors (Å^2^)	
Protein	74.65
Ramachandran (%)	
Favored (%)	96.4
Allowed (%)	3.43
Outlier (%)	0.17
Clash score	9.53
MolProbity score	1.74
MolProbity percentile	100th

a Values in parentheses are for highest-resolution shell.

### αNP-VHH1 binds a variable surface on NP associated with host adaptation.

**FIG 4:**
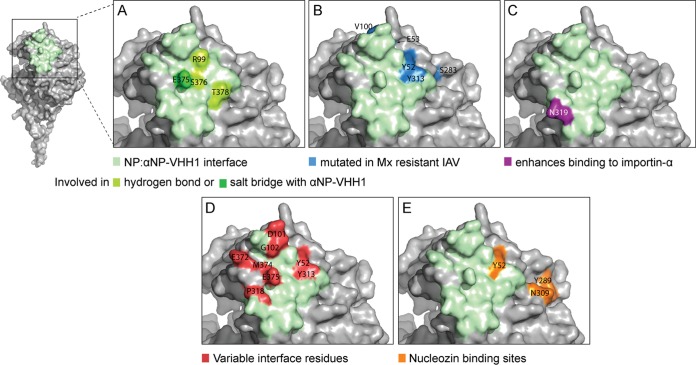
Binding interface of NP and αNP-VHH1. Surface representation of an NP monomer with magnified view of the NP/αNP-VHH1 interface. (A) Residues involved in the interaction that are at least partially occluded upon VHH binding are shown in green. (B) Residues associated with sensitivity of influenza A virus to Mx proteins are shown in blue. (C) The residue that enhances binding to importin-α is shown in purple. (D) Variable interface residues that are part of the largest cluster of variable residues of NP according to the work of Kukol and Hughes (24) are shown in red. (E) The two independent nucleozin binding sites are displayed in orange (34, 50).

We identified the residues involved in the NP/αNP-VHH1 binding interface using PDBePISA (23). The VHH uses residues from all of its three complementary determining regions (CDRs) to interact with NP. On NP, αNP-VHH1 buries a total of 23 residues and an interface of 542 Å^2^ (Fig. 4A). The manner in which the VHH binds NP suggests that it exerts its antiviral properties due to the steric exclusion of potential NP binding partners, including viral or host proteins. The NP binding interface is dispersed over discontinuous elements of secondary structure, which explains why the VHH binds only to correctly folded but not to denatured NP, for example in immunoblotting assays (data not shown). To validate that αNP-VHH1 engages NP at the determined binding site, we generated an E375R mutant of NP, showed that it is expressed to wild-type levels, and confirmed that this mutation in the binding site results in a loss of anti-NP-VHH1 binding (see Fig. S1 in the supplemental material).

To analyze the possible extent of structural conservation of NP at the VHH binding interface, we evaluated conservation grades of the interface residues acquired by large-scale surveys of NP sequences (24). Kukol and Hughes (24) determined conservation grade scores of NP residues using the ConSurf algorithm, which takes into account evolutionary relationships between protein sequences. None of the interface residues were strictly conserved, only 17% were considered conserved, 43% were considered variable, and 39% received scores for an average degree of conservation. Part of the binding interface represents the largest cluster of variable residues on the otherwise rather conserved NP (Fig. 4D). Despite binding to a variable region on NP, we confirmed binding of αNP-VHH1 to the commonly used laboratory strains WSN and PR8 (see Fig. S2 in the supplemental material).

Functional properties of NP are often investigated by mutagenesis or by analysis of naturally occurring, adaptive mutations. In agreement with their variable character, several of the 23 interface residues have been examined in the context of interspecies adaptation. These residues include Asn319, which enhances importin-α affinity (Fig. 4C), and residues Tyr52, Asn101, Tyr313, Glu375, and Ser377, which undergo convergent changes in the process of adaptation from avian to human hosts (8, 25–30). In agreement with the similarity of viral inhibition between αNP-VHH1 and Mx proteins, the αNP-VHH1 binding interface overlaps with a cluster of residues (Tyr52, Glu53, Val100, Ser283, and Tyr313) (Fig. 4B) whose mutation allows escape from Mx proteins (31–33).

Efforts to screen for small-molecule antivirals that perturb the viral replication cycle have yielded a handful of inhibitors that target NP (34, 35). One of them, nucleozin, promotes cytosolic accumulation of incoming vRNPs. Two independent binding sites have been identified for nucleozin (35). One of the two binding sites, at residue Tyr52, overlaps the αNP-VHH1 binding site (Fig. 4E). The second nucleozin binding site, Tyr289/Asn309, is in close proximity to Tyr52 but is not masked by αNP-VHH1 and should therefore be accessible to the drug in the presence of αNP-VHH1. However, the proximity highlights the susceptibility of this NP surface to interference, and αNP-VHH1 may thus also help in better understanding the inhibitory properties of nucleozin and its derivates.

### Effects of αNP-VHH1 on vRNP integrity and nuclear import.

Neither of the two characterized NLSs is in proximity to the αNP-VHH1 binding site (Fig. 3C and D). Nuclear import of NP alone is unaffected by αNP-VHH1, while nuclear import of incoming vRNPs is strongly inhibited (16, 17). Thus, the structure of the vRNP complex must be important for αNP-VHH1 to elicit its inhibitory function.

**FIG 5:**
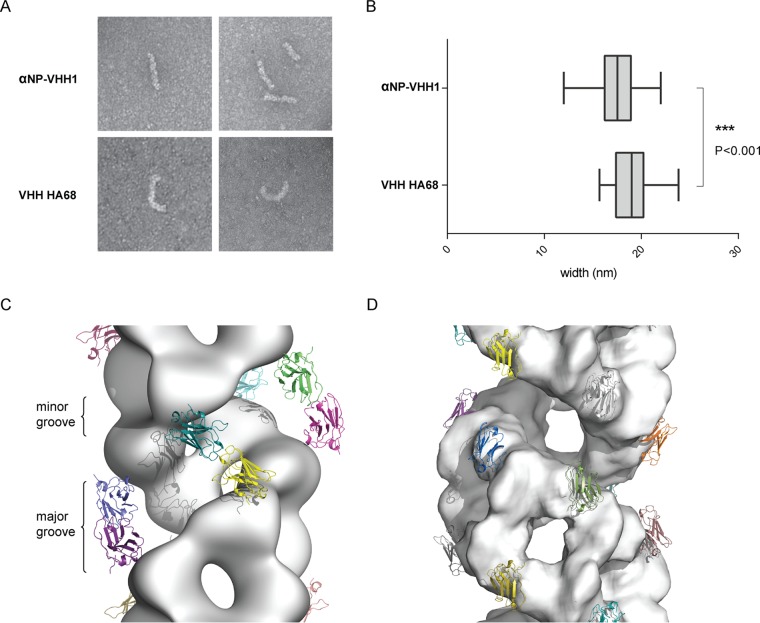
αNP-VHH1 binding to vRNPs. (A) vRNPs purified from influenza virus A/PR/8/34 virions were treated with an excess of αNP-VHH1 or a control VHH (VHH HA68 against IAV HA [51]) and visualized by negative-stain electron microscopy. (B) The width of vRNPs (*n* = 28) was measured at two positions using ImageJ. A two-tailed Student *t* test was performed to evaluate statistical significance. (C and D) Superposition of the NP/αNP-VHH1 structure on vRNP models from the work of Moeller et al. (38) (PDB ID 2YMN) (C) and Arranz et al. (37) (PDB ID 4BBL) (D). Electron density is shown for both models, and colored VHHs are located according to the NP orientation in the model.

To evaluate the general effect of αNP-VHH1 binding to vRNPs, we purified vRNPs from virions, added an excess of αNP-VHH1 or a control VHH, and analyzed the appearance of vRNPs by negative-stain electron microscopy (EM). We did not detect gross differences between vRNPs complexed with anti-NP-VHH1, indicating that αNP-VHH1 does not disrupt the overall structure of the complex (Fig. 5A). A typical VHH is 2 by 3.5 nm in size (36). Assuming that the VHH binds to the periphery of the vRNP, one would expect a slight increase of vRNP width. However, vRNPs examined in the presence of αNP-VHH1 exhibit a slightly reduced width (Fig. 5B). Purified vRNPs often adopted a slightly curved shape. Upon addition of αNP-VHH1, we noted a trend for vRNPs to adopt curved shapes less frequently, but we were unable to validate this tendency statistically.

To analyze the possible impact of αNP-VHH1 binding to vRNPs on a structural level, we examined the two available cryo-EM models of vRNPs (37, 38). While overall similar, the two models differ in NP orientation and handedness. The binding interface for αNP-VHH1 is exposed in both models and should thus allow VHH binding. The right-handed helical model of Moeller et al. is based on vRNPs purified from transiently transfected cells transcribing influenza virus genome segments and expressing NP and polymerase subunits (38). When we superimpose our structure on this model, αNP-VHH1 blocks the major groove of the vRNP complex (Fig. 5C), which would reduce the accessible surface of the vRNP and thus limit interactions of the vRNP with other viral and host proteins.

The vRNPs that were used for the left-handed helical model of Arranz et al. (37) derive from purified virions and therefore more likely represent the incoming vRNPs encountered by anti-NP-VHH1 in the cytosol. Modeled onto this structure, anti-NP-VHH1 is positioned on the edge of the major groove without blocking it (Fig. 5D). Instead, αNP-VHH1 slightly clashes with the head domain of the adjoining NP molecule. Considering the inherent structural flexibility of the NP tail loop and thus of vRNPs, this clash may not be all that detrimental. However, the necessary structural compensation might explain the slightly thinner vRNPs observed by EM and why some vRNPs appear less curved. Importantly, according to this model, αNP-VHH1 masks NLS2 of the adjacent NP protomer, providing a plausible explanation for the inhibition of vRNP nuclear import.

## DISCUSSION

To identify new vulnerabilities in the life cycle of influenza A virus, we used cytosolically expressed single-domain antibodies, also called VHHs, that target the nucleoprotein (NP). To relate the inhibitory properties of αNP-VHH1 to the structural features that it recognizes on NP, we determined the crystal structure of the VHH in complex with NP. The binding site of αNP-VHH1 overlaps evolutionarily variable residues implicated in interactions with host proteins, including both a supporting (importin-α) and an antagonizing (Mx proteins) host factor. αNP-VHH1 exploits a surprisingly similar vulnerability as do the host’s antiviral, interferon-induced Mx proteins.

The antiviral activity of Mx GTPases has long been recognized, first described as a restriction factor for influenza A virus (39). While there is consensus on the general antiviral activity of MxA/Mx1 as an entity that targets both the viral PB2 and NP, the exact mode of action has remained elusive. Several residues on NP alter viral susceptibility to Mx1 proteins, some of which are occluded by αNP-VHH1. Whether these residues directly alter affinity to Mx proteins has so far not been shown, and no structure of an Mx protein in complex with NP has been reported. Because of possible alternative binding sites and affinities of Mx to monomeric NP versus NP assembled in vRNPs, these interactions might be difficult to show unambiguously. Our data show that occluding this surface on the NP body domain is sufficient to mimic the antiviral activity of Mx GTPases, suggesting that a direct interaction of Mx proteins with this surface is likely. The involvement of (an) additional, unknown factor(s) that contribute(s) to Mx activity cannot be excluded. However, we can conclude that targeting NP alone, and not PB2, is sufficient to mimic the effect of Mx proteins. Because of the structurally defined αNP-VHH1 binding site on NP, it is the ideal model protein to investigate possible antiviral mechanisms of Mx proteins.

Antiviral effects of Mx are dependent on GTPase activity in most settings (40). Mx proteins are known to form ring-like structures that—in the case of MxA—are thought to clasp around the incoming vRNPs to prevent nuclear import (41). Our VHHs lack any enzymatic activity and do not oligomerize, suggesting that high-affinity binding may be sufficient for antiviral activity. Oligomerization and the resulting avidity effects may allow the same antiviral mechanism at lower-affinity interactions between NP and Mx proteins. In the light of evolution, this may be advantageous for two reasons: (i) it allows a single Mx homolog to act as a potent broad-spectrum effector that binds to multiple virus families with lower affinity and (ii) if low-affinity binding is sufficient for antiviral activity, then it would be more difficult to escape from all Mx activity by antigenic drift.

Superposition of αNP-VHH1 on EM-based vRNP models showed that αNP-VHH1 could mask the NLS2 on adjoining NP molecules. Mx proteins might function in a similar fashion and prevent vRNP interactions with host factors, including those important for nuclear import. Indeed, a crucial residue for host specificity that enhances binding to importin-α, Asn319, is found in the interface with αNP-VHH1. In addition, escape from Mx proteins with mutations in residues Val100, Ser283, and Tyr313 occurs at the expense of nuclear import efficiency (32).

Why this surface is important for nuclear import of vRNPs is unknown. Modeled on the vRNP structure from the work of Arranz et al. (37), our data showed the proximity of αNP-VHH1 to the NLS2 on an adjoining NP molecule, suggesting that VHH binding to vRNPs prevents importin binding to NLS2. It is conceivable that importin-α, when bound specifically to one NLS of NP, is stabilized by residues of the αNP-VHH1 interface, including Val100, Ser283, Tyr313, and Tyr319. However, the inherent flexibility and heterogeneity of the vRNP complexes present a major challenge to prove this contention by structural means. Higher-resolution structures of the vRNP complex, ideally complexed with host factors, are needed. The structure of anti-NP-VHH1 with NP might be of use in determining the exact orientation of NP and handedness of the helix in such higher-order complexes by yielding more static templates for analysis.

The strength of intracellular VHHs as specific perturbants of protein function is their ability to mask specific structurally defined NP epitopes without prior manipulation of the virus by mutation. This approach is inherently limited by the ability of the virus to shut down host protein synthesis, including that of inhibitory VHHs. While we were able to circumvent these limitations using inducible VHH expression to some extent, cell-penetrating VHHs or other means of permeabilization that allow efficient delivery from extracellular space could also find application (42, 43). Such an approach would allow an evaluation of the importance of the blocked surface at later stages of infection and, importantly, transform VHHs into discovery tools for antiviral agents suitable for therapeutic intervention.

Given the continued relevance of influenza virus as a serious health threat and its ability to rapidly acquire resistance against drugs or escape from immune responses that target HA, NA, and M2, the more conserved influenza virus proteins, including NP, may prove to be alternative targets for intervention. So far, we have developed more than 20 IAV NP-specific VHHs that bind to at least four unique binding sites on NP (16, 17). Continued efforts in this direction might help to map more precisely the contributions of different NP surfaces to the influenza virus life cycle and inspire the development of novel antivirals. In conclusion, the crystal structure of an inhibitory VHH in complex with NP uncovers a new vulnerability in the virus life cycle and may phenocopy mechanisms of actions of the cellular antiviral defense. The ease of expression and the capability of binding to common laboratory-adapted influenza virus strains, combined with our detailed molecular characterization of the binding site, make αNP-VHH1 a versatile tool to identify the specific contributions of this NP surface and of host factors that potentially compete with αNP-VHH1 for binding.

## MATERIALS AND METHODS

### Virus.

Influenza A/WSN/33 virus used for infection experiments was propagated and titrated on MDCK cells. Infections were performed in Dulbecco modified Eagle medium (DMEM) with 0.3% bovine serum albumin (BSA) for 1 h at 37°C. vRNPs were purified from influenza virus A/PR/8/34 virions purchased from Charles River Laboratories.

### Cell lines.

Human HEK 293T cells and dog MDCK cells were obtained from ATCC and grown in DMEM with 10% fetal bovine serum (FBS). The A549 cell line inducibly expressing αNP-VHH1-HA, derived from A549 cells obtained from ATCC, was described earlier (16) and was cultivated in DMEM with 10% FBS and 500 μg/ml G418.

### Reagents.

Doxycycline hyclate (Dox) was purchased from Sigma-Aldrich. Nickel-nitrilotriacetic acid (NTA) beads were purchased from Qiagen. Mouse anti-HA.11 (clone 16B12) coupled to Alexa Fluor (AF) 488 was purchased from Life Technologies. Mouse anti-HA.11 (clone 16B12) was acquired from BioLegend. VHHs coupled to either Alexa Fluor (AF) 647 or 5-carboxytetramethylrhodamine (TAMRA) were generated using sortase A as described earlier (44). Hybridoma cells secreting mouse monoclonal anti-IAV NP (clone H16-L10-4R5; ATCC HB-65) were obtained from ATCC, and antibodies in the supernatant were purified using a protein G column.

### Infection assay.

To analyze the effect of αNP-VHH1 expressed at different times during infection, A549 cells inducibly expressing the VHH were infected with A/WSN/33 at a multiplicity of infection (MOI) of 1. VHH expression was induced at the indicated time points relative to infection with 1 μg/ml doxycycline (final concentration). Six hours postinfection, cells were trypsinized, fixed in 4% paraformaldehyde (PFA), permeabilized with 0.1% saponin, and stained with anti-HA-AF488 and αNP-VHH2-AF647. Fraction of infected cells (NP) and geometric mean fluorescence intensity (VHH-HA) were quantified by flow cytometry using a BD Accuri cytometer and the FlowJo software package.

### Titration of released virus.

To quantify release of progeny virus from cells expressing αNP-VHH1, A549 cells inducibly expressing the VHH were infected with A/WSN/33 at an MOI of 1 in the presence of tosylsulfonyl phenylalanyl chloromethyl ketone (TPCK) trypsin. VHH expression was induced at the indicated time points relative to infection with 1 μg/ml doxycycline (final concentration). Twenty-four hours postinfection, supernatants were collected, filtered, diluted in a 2-fold dilution series, and used to infect MDCK cells for 1 h at 37°C. The inoculum was replaced with DMEM, 0.35% BSA, and 1.5% carboxymethyl cellulose in the absence of trypsin to avoid spreading of the virus. After 24 h, cells were fixed in 4% PFA, permeabilized with 0.1% saponin in phosphate-buffered saline (PBS)–2% BSA, and stained with 4′,6-diamidino-2-phenylindole (DAPI) and αNP-VHH2-TAMRA. Fluorescence images of the MDCK monolayer were acquired using a Cytation 3 cell imaging multimode reader (BioTek); infected cells (nuclei) were quantified using CellProfiler (45).

### Influenza virus polymerase reconstitution assay.

To assess influenza virus polymerase activity, 293T cells were transiently transfected with 200 ng of pCAGGS NP and 50 ng of pCAGGS PB1, pCAGGS PB2, and pCAGGS PA. To provide an artificial genome segment, 50 ng of pPolI-RT plasmid was cotransfected, from which an artificial genome segment was transcribed that contained the NA untranslated regions and encoded either EGFP or mCherry-2TA-EGFP. One hundred fifty nanograms of pCAGGS vector encoding αNP-VHH1, VHH7 (anti-murine-MHCII), or pCDNA3.1 encoding MxA or Mx1 was additionally cotransfected where indicated. All transfections were performed using Lipofectamine 2000. Twenty-four hours posttransfection, cells were trypsinized and the fraction of EGFP-positive cells was quantified by flow cytometry using a BD Accuri cytometer.

### Protein expression and purification.

The sequence encoding αNP-VHH1 with a C-terminal sortase recognition site (LPETG) followed by a His tag was cloned into a pHEN6 expression vector for periplasmic expression. *Escherichia coli* WK6 bacteria were transformed with the vector, and expression was induced with 1 mM isopropyl-β-d-thiogalactopyranoside (IPTG) at an optical density at 600 nm (OD_600_) of 0.6; cells were grown overnight at 30°C. The VHH was retrieved from the periplasm by osmotic shock and purified by Ni-NTA affinity purification and size exclusion chromatography on a Superdex 75 column.

NP from influenza virus A/WSN/33 with a C-terminal His tag was cloned into the pET30 expression vector. *E. coli* BL21(DE3) bacteria were transformed and grown in Terrific Broth at 37°C until reaching an OD_600_ of 0.5 and at 25°C until reaching an OD_600_ of 0.6. Protein expression was induced with 1 mM IPTG at an OD_600_ of 0.6, and cells were grown for an additional 3 h at 25°C. Bacterial pellets were resuspended in 25 mM Tris-HCl, pH 7.5, 1 M NaCl, 0.2% NP-40, 10 units/ml Benzonase, and 0.1 mg/ml lysozyme. Cells were lysed by sonication, and NP was purified on Ni-NTA agarose, Mono S ion exchange, and Superdex 200 size exclusion columns.

### Crystallization.

For cocrystallization, purified αNP-VHH1 was mixed in a 3:1 molar ratio with recombinant NP and purified by size exclusion on a Superdex 200 column. Both tri- and monomeric peaks were collected, and VHH binding was confirmed by SDS-PAGE and Coomassie blue staining. The complex was concentrated to 4 mg/ml in 20 mM Tris-HCl, pH 7.5, 200 mM NaCl buffer using a protein concentrator. Initial crystal growth was observed in 0.1 M sodium acetate-1.5 M ammonium sulfate in a vapor diffusion experiment in a 96-well sitting drop setup (Procomplex; Qiagen) at 18°C. Crystal growth was optimized with 0.025% (vol/vol) dichloromethane, and diffraction-quality crystals were grown in a 24-well vapor diffusion hanging drop setup. Crystals were cryoprotected in 20% glycerol and flash frozen in liquid nitrogen.

### Data processing and structure determination.

Data sets were collected at the Advanced Photon Source user end station 24-IDC. Data reduction was performed in HKL2000 (46). Molecular replacement (MR) was performed in the PHENIX suite using PhaserMR (47). As an MR model for NP, we used PDB identifier (ID) 2IQH (NP) (1) and PDB ID 4KRL (VHH) (21). Refinement was performed using phenix.refine, and the model was built in Coot (48).

### Purification of vRNPs and electron microscopy.

vRNPs were isolated and purified from IAV PR8 virions as described elsewhere (49). In brief, influenza virus A/PR/8/34 was concentrated and virions were lysed in 100 mM KCl, 5 mM MgCl_2_, 5% (wt/vol) glycerol, 50 mM octylglucoside, 10 mg/ml lysolecithin, 1.5 mM dithiothreitol, 100 mM morpholineethanesulfonic acid (MES), pH 5.5. vRNPs were then separated from other viral proteins on a glycerol gradient and concentrated. vRNPs were treated with an excess of αNP-VHH1 or the control VHH in 50 mM Tris-HCl, pH 7.5, 150 mM NaCl and subsequently stained with 2% uranyl acetate. Electron micrographs were recorded with a FEI Tecnai Spirit Bio-Twin microscope, and images were analyzed with ImageJ.

### LUMIER assay.

To analyze binding of VHHs to wild-type and mutant versions of IAV NP, we applied LUMIER assays as described in detail before (17). 293T cells were cotransfected with pCAGGS anti-NP-VHH1-HA and wild-type or mutant E375R pEXPR NP-Renilla (derived from NP of influenza virus A/WSN/33) or pEXPR VSV-N-Renilla using Lipofectamine 2000. Twenty-four hours posttransfection, cells were lysed and incubated in Lumitrac 600 96-well plates (Greiner) coated with anti-HA.11 antibody to capture the VHH. Activity of the copurified luciferase was quantified by addition of coelenterazine-containing *Renilla* luciferase substrate mix (BioLux Gaussia luciferase assay kit; New England BioLabs), and light emission was measured using a SpectraMax M3 microplate reader (Molecular Devices).

## SUPPLEMENTAL MATERIAL

Figure S1  NP mutation E375R abolishes αNP-VHH1 binding. αNP-VHH1 and *Renilla* luciferase fusions of influenza virus A/WSN/33 NP wild type (WT) or E375R or vesicular stomatitis virus N were transiently coexpressed in 293T cells. Cell lysates were incubated in 96-well plates coated with anti-HA antibody to capture the VHHs. (A) Activity of the copurified luciferase was measured. Emitted light was normalized to luciferase activity in the lysate. (B) *Renilla* activity of the *Renilla*-NP/N fusion proteins in cell lysates shown as relative light units (RLU) emitted. Data in panels A and B are from three independent experiments (± standard errors of the means). (C) Lysates from cells transfected as described above were subjected to immunoblot analysis using anti-NP, anti-glyceraldehyde-3-phosphate dehydrogenase, and anti-HA tag (VHH-HA) antibodies. Download Figure S1, TIF file, 0.8 MB

Figure S2  αNP-VHH1 stains NP in cells infected with WSN and PR8 strains. A549 cells were infected with influenza virus A/WSN/33 or A/PR/8/34 and harvested 6 h postinfection. Cells were fixed, permeabilized, stained with αNP-VHH1-Alexa Fluor 647, and analyzed by flow cytometry. Download Figure S2, TIF file, 0.2 MB
