# Supplementary figures and images for: The Antiviral Mechanism of an Influenza A Virus Nucleoprotein-Specific Single-Domain Antibody Fragment
Source: mBio. 2016 Dec 13;7(6):e01569-16. doi: 10.1128/mBio.01569-16 (PMC5156300; doi:10.1128/mBio.01569-16)

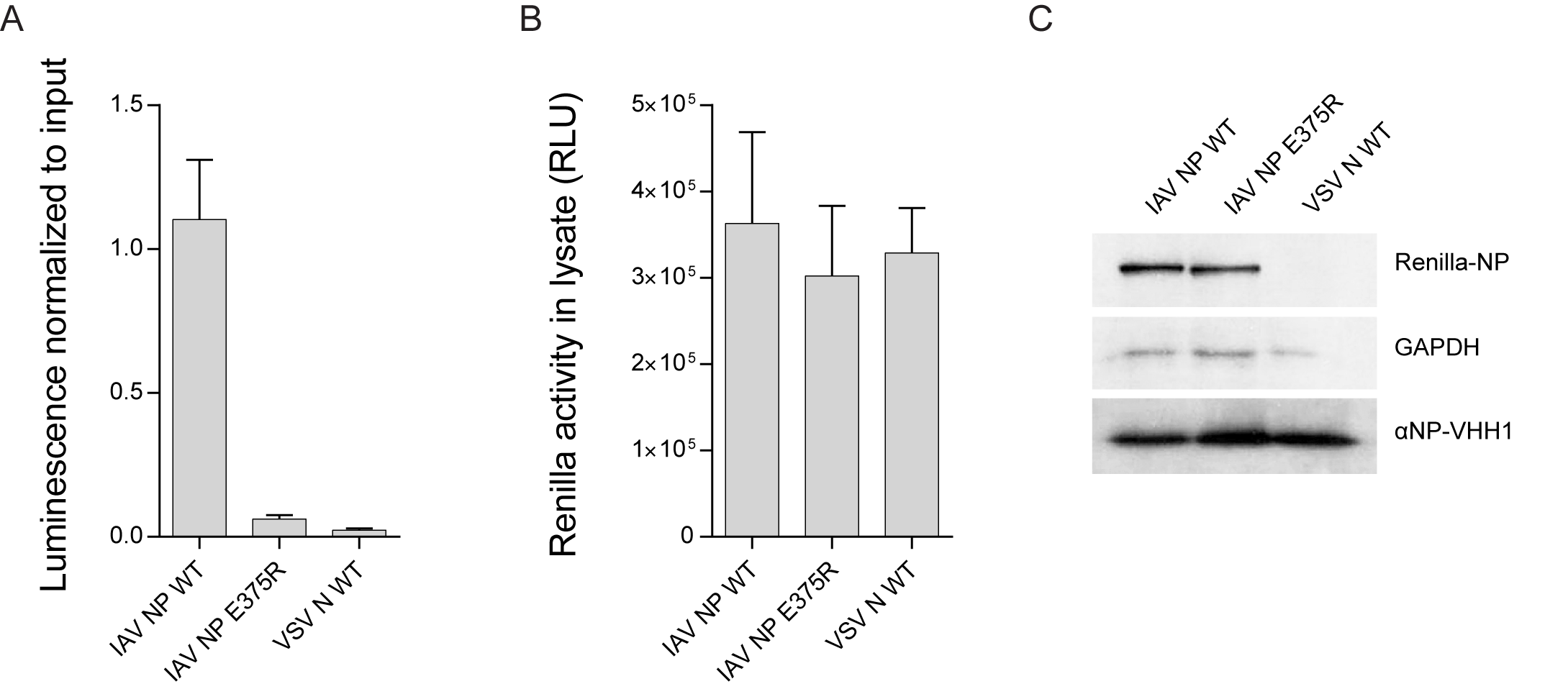

Supplement: Figure S1 — NP mutation E375R abolishes αNP-VHH1 binding. αNP-VHH1 and Renilla luciferase fusions of influenza virus A/WSN/33 NP wild type (WT) or E375R or vesicular stomatitis virus N were transiently coexpressed in 293T cells. Cell lysates were incubated in 96-well plates coated with anti-HA antibody to capture the VHHs. (A) Activity of the copurified luciferase was measured. Emitted light was normalized to luciferase activity in the lysate. (B) Renilla activity of the Renilla-NP/N fusion proteins in cell lysates shown as relative light units (RLU) emitted. Data in panels A and B are from three independent experiments (± standard errors of the means). (C) Lysates from cells transfected as described above were subjected to immunoblot analysis using anti-NP, anti-glyceraldehyde-3-phosphate dehydrogenase, and anti-HA tag (VHH-HA) antibodies. Download [file mbo006163103sf1.tif]

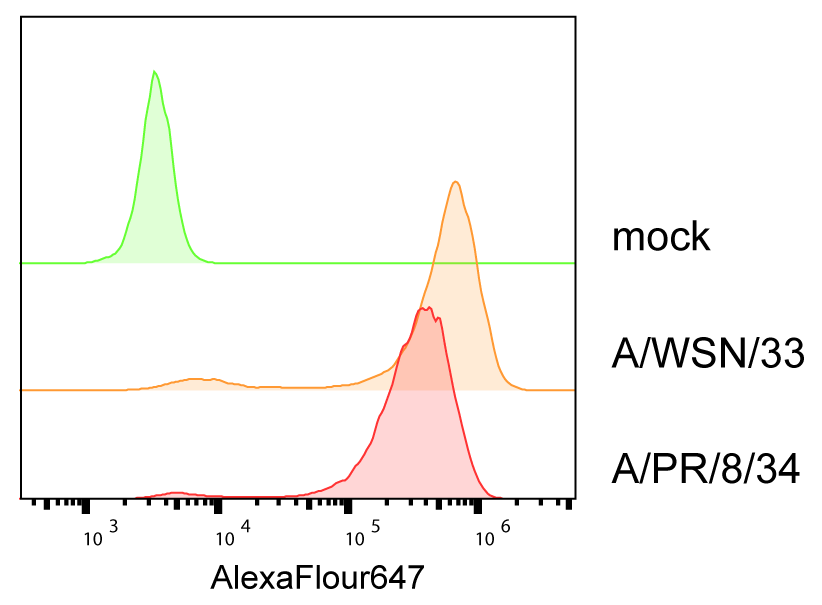

Supplement: Figure S2 — αNP-VHH1 stains NP in cells infected with WSN and PR8 strains. A549 cells were infected with influenza virus A/WSN/33 or A/PR/8/34 and harvested 6 h postinfection. Cells were fixed, permeabilized, stained with αNP-VHH1-Alexa Fluor 647, and analyzed by flow cytometry. Download [file mbo006163103sf2.tif]
